# Failure to Respond to Food Resource Decline Has Catastrophic Consequences for Koalas in a High-Density Population in Southern Australia

**DOI:** 10.1371/journal.pone.0144348

**Published:** 2016-01-06

**Authors:** Desley A. Whisson, Victoria Dixon, Megan L. Taylor, Alistair Melzer

**Affiliations:** 1 Centre for Integrative Ecology, School of Life and Environmental Sciences, Deakin University, Burwood, Victoria, Australia; 2 School of Medical and Applied Sciences, Central Queensland University, Rockhampton, Queensland, Australia; University of Southern Queensland, AUSTRALIA

## Abstract

Understanding the ability of koalas to respond to changes in their environment is critical for conservation of the species and their habitat. We monitored the behavioural response of koalas to declining food resources in manna gum (*Eucalyptus viminalis*) woodland at Cape Otway, Victoria, Australia, from September 2011 to November 2013. Over this period, koala population density increased from 10.1 to 18.4 koalas.ha^-1^. As a result of the high browsing pressure of this population, manna gum canopy condition declined with 71.4% manna gum being completely or highly defoliated in September 2013. Despite declining food resources, radio collared koalas (N = 30) exhibited high fidelity to small ranges (0.4–1.2 ha). When trees became severely defoliated in September 2013, koalas moved relatively short distances from their former ranges (mean predicted change in range centroid = 144 m) and remained in areas of 0.9 to 1.0 ha. This was despite the high connectivity of most manna gum woodland, and close proximity of the study site (< 3 km) to the contiguous mixed forest of the Great Otway National Park. Limited movement had catastrophic consequences for koalas with 71% (15/21) of radio collared koalas dying from starvation or being euthanased due to their poor condition between September and November 2013.

## Introduction

Conservation of a species relies on an understanding of how it responds to habitat change or loss [1–3]. This is particularly important for species that have highly specialised dietary or nesting/roosting requirements and limited ability to adapt to modified habitats. Where such species occur in landscapes subject to a change in resource distribution and availability, survival of individuals relies on their ability to relocate to new habitats [4]. However, this may be limited by landscape factors such as the availability and distance to suitable habitat patches, and the density of populations already in the alternate habitat patches; and the physical and behavioural characteristics of the species [1–3].

Most studies of the behavioural response of a species to habitat change have been undertaken where logging or bushfire are the cause of habitat disturbance (e.g. [2,3,5,6]). These studies suggest that disturbance may have little impact on space use by individuals, presumably due to high site fidelity [2,3,6]. Although remaining in disturbed areas may not affect survival of highly mobile and generalist herbivores [2,7], this behaviour may have dire consequences for other species. For example, strong site fidelity of tree kangaroos (*Dendrolagus lumholtzi*), resulted in individuals remaining in a cleared rainforest fragment (surviving for a short period in the ground debris) and ultimately suffering high predation rates [3]. Similarly, one study observed high mortality in greater gliders (*Petauroides volans*) following forest clearing [6]. Most gliders only moved as their nest trees were being felled, and failed to relocate successfully to neighbouring habitat.

An understanding of the behavioural response of koalas (*Phascolarctos cinereus*) to changing habitats is becoming more important as habitat loss, fragmentation and degradation increase throughout the species’ range [8]. The koala is a specialist folivore that subsists on a nutrient-poor diet of *Eucalyptus* foliage [9,10]. Its preference for *Eucalyptus* species that are relatively high in nitrogen and low in concentrations of plant secondary metabolites is a major factor defining habitat quality and the distribution of koalas in a landscape [10–12]. As a consequence of its low-energy diet and patchy distribution of preferred food trees, the koala may be particularly vulnerable to changing landscapes and availability of preferred food trees (e.g. [13]).

Although habitat clearance for urban or industrial development has been implicated as the major cause of habitat loss throughout the koala’s range [8], in some localised areas of southern Australia, the unsustainable browsing pressure of high density populations of koalas also negatively impacts their food availability [14, 15]. In several locations in Victoria and South Australia, this has resulted in the localised loss of preferred food tree species and a subsequent decline in the koala population (see [15] for review). Where these situations occur in landscapes where alternative but less preferred habitats are available, there is an opportunity to examine the behavioural response of koalas to declining availability of food resources. This is the context of the current study which was undertaken at Cape Otway, Victoria, over a two-year period encompassing an increase in koala population density, and a decline in food availability that ultimately resulted in a collapse of the koala population. Specifically, we aimed to determine: (i) home range size and site fidelity of koalas in a high-density population, and (ii) the influence of the decline in food resources on movements and survival of koalas.

## Methods

### Ethics statement

This study was approved by the Deakin University Animal Ethics Committee (A01-2011) and conducted under permit (10005379) by the Victorian Department of Environment, Land, Water and Planning. Permission to access private property was obtained from landholders C. and P. Marriner.

### Study area

**Fig 1:**
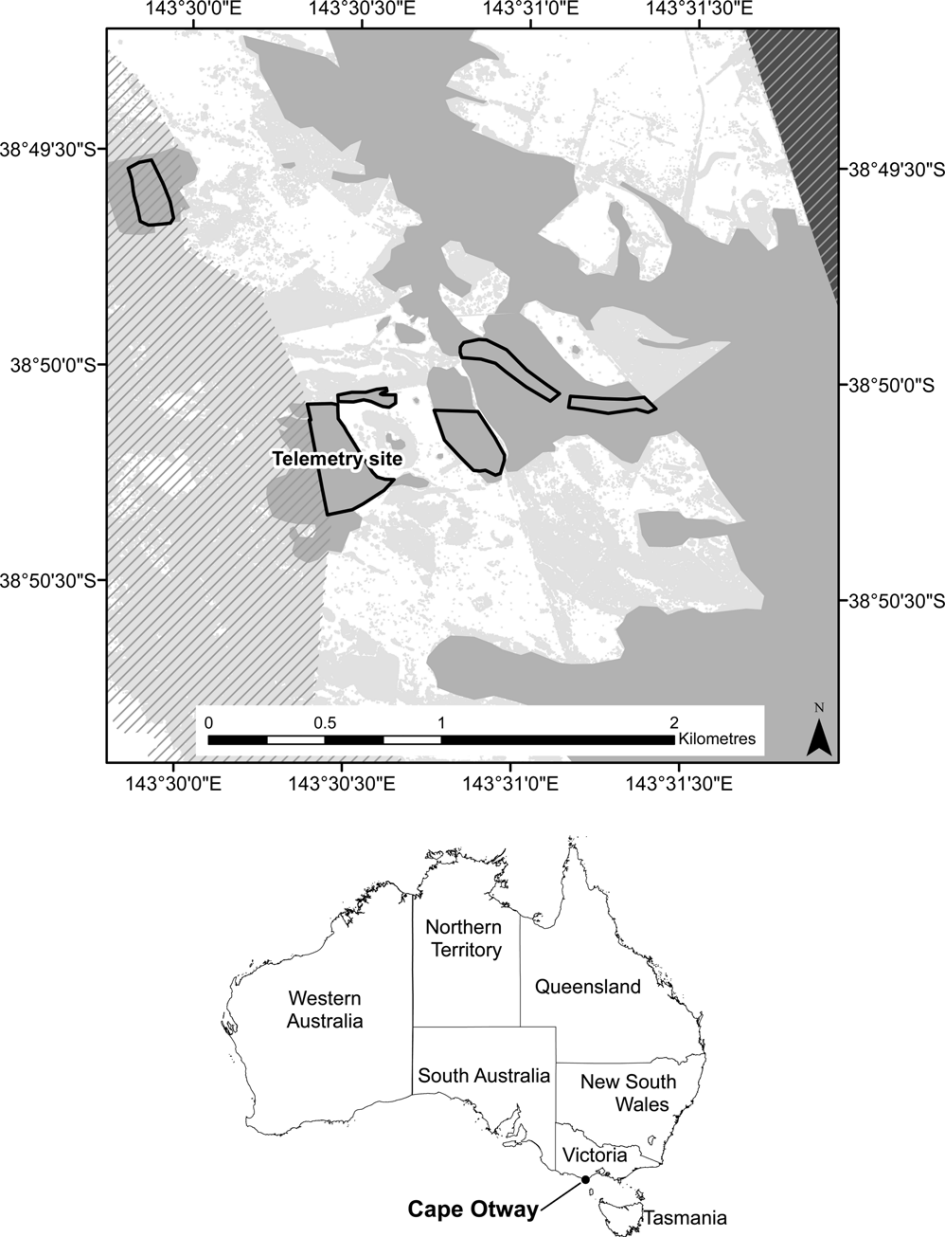
The study area in Victoria, Australia. The hatched areas are part of the Great Otway National Park. Vegetation types are coastal shrubland (light grey) manna gum woodland (medium grey), and mixed woodland (dark grey). Sites are outlined with a solid line.

The study was undertaken from September 2011 to November 2013 in manna gum woodland at Cape Otway (38°50’06”S, 143°30’25”E), Victoria, Australia (Fig 1). The area comprises a mosaic of manna gum woodland, coastal shrubland, and cleared pasture. It is bordered by tall mixed forests in the north and east and coastal heathland in the south and west. Average annual rainfall is 898 mm and temperatures range from a mean monthly minimum of 7.5°C in July to a mean monthly maximum of 21.6°C in February (Bureau of Meteorology, long-term averages, Station ID 090015).

### Koala populations and tree canopy condition

Six sites in manna gum woodland (Fig 1) were surveyed for koalas on one day in September each year. This month was chosen for providing the best estimate of the adult population. At this time, most young are small and easily identified as being dependent. In each assessment, up to eight observers systematically searched all trees in each site and recorded the number of koalas sighted. The sex of each koala and the presence of dependent young with adult females were recorded.

Immediately following the koala surveys, the canopies of 20 to 40 randomly selected manna gums in each site were assessed. For each tree, an index of canopy condition was recorded as one of four classes: 1 = completely defoliated; 2 = highly defoliated; 3 = some defoliation; 4 = very little defoliation. Assessments were conducted by multiple trained observers provided with reference photos of representative trees of each condition class.

### Telemetry

A combination of radio tracking and GPS data logging was used to obtain locations for sexually-mature koalas (>6 kg [16]) in one manna gum site over the study period. The study period encompassed three breeding (September–February) and two non-breeding (March–August) seasons. A total of 30 (15 males, 15 females) adult koalas were used over this period although the number sampled in each season varied between 11 and 28. Twenty individuals were sampled in at least two seasons of the study.

Koalas were captured using a standard noose and flag technique (see [17]). At first capture, each koala was marked with a uniquely numbered ear-tag and fitted with a VHF radio collar (Sirtrack Pty Ltd, Havelock North, New Zealand) modified to incorporate a rubber weak link. A GPS data logger (i-gotu GT-120, Mobile Action Technology, www.i-gotu.com), removed from its casing and encapsulated in heat-shrink tubing for waterproofing, was attached with electrical tape to the top of each collar. GPS data loggers were programmed to record locations at 90-minute intervals which allowed each koala’s locations to be recorded for an average of 31.3 ° 0.68 days (hereafter a ‘GPS sample period’). Sex and age class (from tooth wear [18]), weight (kg), muscle condition (scored 1 to 10 [19]), and presence of dependent young were recorded. Koalas were released at their points of capture.

When possible, each koala was recaptured once during each subsequent season and the GPS data logger replaced. Koala weight, muscle condition, and presence of dependent young were again recorded prior to the koala being released at its point of capture. At one to two week intervals throughout the study, radio collared koalas were located by tracking on foot with a Telonics TR4 receiver (Telonics Inc, Mesa, AZ, USA) and a hand-held 3-element Yagi antenna.

### Data analysis

A generalised linear mixed model (GLMM) using a Gaussian distribution was used to examine changes in koala population density between years. Site was included as a random term. Model fit was validated through inspection of residuals.

To determine the validity of home range estimation, within-sample site fidelity for each radio collared koala was identified using two methods: 1) mean squared distance from the centre of activity (MSD [20]); and 2) a linearity index (LI [21], the distance between the endpoints of an animal’s path divided by the total distance travelled). For each individual, 100 movement trajectories were simulated using the same number of relocations as the real data but with random turn angles between relocations [22]. The MSD and LI of simulated trajectories were compared with those of the real trajectory. Site fidelity was presumed to exist and home range estimation valid [22,23] when both the MSD and LI of an individual were lower than those of the simulated trajectories [22,24,25].

Home range was estimated for each GPS sample period when within-sample site fidelity according to the preceding criteria was indicated. Fixed-kernel analysis with a plug-in bandwidth [26] was used to estimate total ranges (95% isopleths). This approach was considered suitable to examine seasonal use of habitat by a species with relatively low mobility in a small geographic area, and for data that was spatially autocorrelated [26].

GLMMs were used to examine the influence of gender and season (breeding or non-breeding) effects on total ranges. Both the interaction of these effects and their individual contributions were included in the model. Because some individuals were sampled in multiple seasons, a unique identifier was assigned to each koala and included as a random term in the model. Data were log-transformed to approximate a normal distribution and model fit was validated through inspection of residuals.

Inter-season site fidelity was examined by calculating a Utilisation Distribution Overlap Index (UDOI [27]) between consecutive GPS sampling periods for each animal for which data was available. The distance between consecutive total range centroids also was determined. GLMMs were used to examine distance between range centroids over the study period. A unique identifier for each koala was included as a random term.

Home ranges were estimated in Geospatial Modelling Environment (GME [28]). UDOI was determined in ArcMAP 10.2.2 [29]. Other analyses were conducted in R [30] using packages lme4 (linear mixed effects models [31]) and rhr (site fidelity [23]).

## Results

### Koala populations and tree canopy condition

Koala populations increased each year from a mean of 10.1 koalas.ha^-1^ in 2011 to 18.4 koalas.ha^-1^ in 2013 (Table 1). The proportion of females observed with dependent young during surveys was 54% and did not vary between years (Table 1). In the telemetry study, 80% (4/5) and 76% (10/13) of females produced young in the 2011 and 2012 breeding period respectively. High mortality of study animals during the 2013 breeding period precluded an estimate of fecundity for that period. Tree canopy condition declined over the study period (Table 2), with 71% of trees completely or highly defoliated in September 2013.

**Table 1:** Characteristics of koala populations in six manna gum woodland sites assessed in September 2011–2013.

Year	Mean density (koalas.ha^-1^) ± *SE*	% females	% females with young	Total number of koalas observed
**2011**	10.1 ° 1.2	51.8	55.1	183
**2012**	14.2 ° 2.0	57.7	64.4	221
**2013**	18.4 ° 2.5	62.4	42.7	269
**Statistics**^**a**^	2012: t_8.907_ = 1.67, *P* = 0.129; 2013: t_9.355_ = 3.33, *P* = 0.008	$\chi_{2}^{2}$ = 8.11, *P* = 0.017	$\chi_{2}^{2}$ = 4.77, *P* = 0.09	

^a^The reference category for year in GLMMs was 2011.

**Table 2:** Percentage of trees from six manna gum woodland sites in each canopy condition class September each year from 2011 to 2013. Classes are: 1 = completely defoliated; 2 = highly defoliated; 3 = some defoliation; 4 = very little defoliation.

Year	Class 1	Class 2	Class 3	Class 4	Number of trees assessed
2011	7.6%	29.4%	39.1%	23.9%	184
2012	10.7%	32.1%	45.0%	12.1%	140
2013	12.2%	59.2%	19.4%	9.2%	98

### Home range and movements

Within-sample site fidelity was indicated in 93.5% (72/77) of GPS sampling periods. Site fidelity was not indicated for one male in the 2011–2012 breeding season and 2012 non-breeding season, and for one female that undertook a long-distance movement incorporating some of each of the non-breeding and breeding seasons in 2012, and another long-distance movement in the 2013 breeding season. Total home ranges (K95) based on GPS sampling periods (when within-sample site fidelity was indicated) generally were representative of individuals’ ranges in each season. Radio tracking locations for individuals from an entire season were inside or within 25 m of the total range estimated for 87.5% (63/72) of GPS sampling periods. For 12.5% (9/72; 5 males, 4 females) of GPS sampling periods, radio tracking showed that individuals temporarily (one week duration) moved up to 140 m away from their total ranges outside of the GPS sampling period. Additionally, GPS sampling periods did not completely capture two of the three long-distance, round-trip movements by three females. These movements were 475 to 1600 m from each individual’s former range and lasted 30 to 121 days. These females only undertook long-distance movements once in the two years that each was monitored.

**Fig 2:**
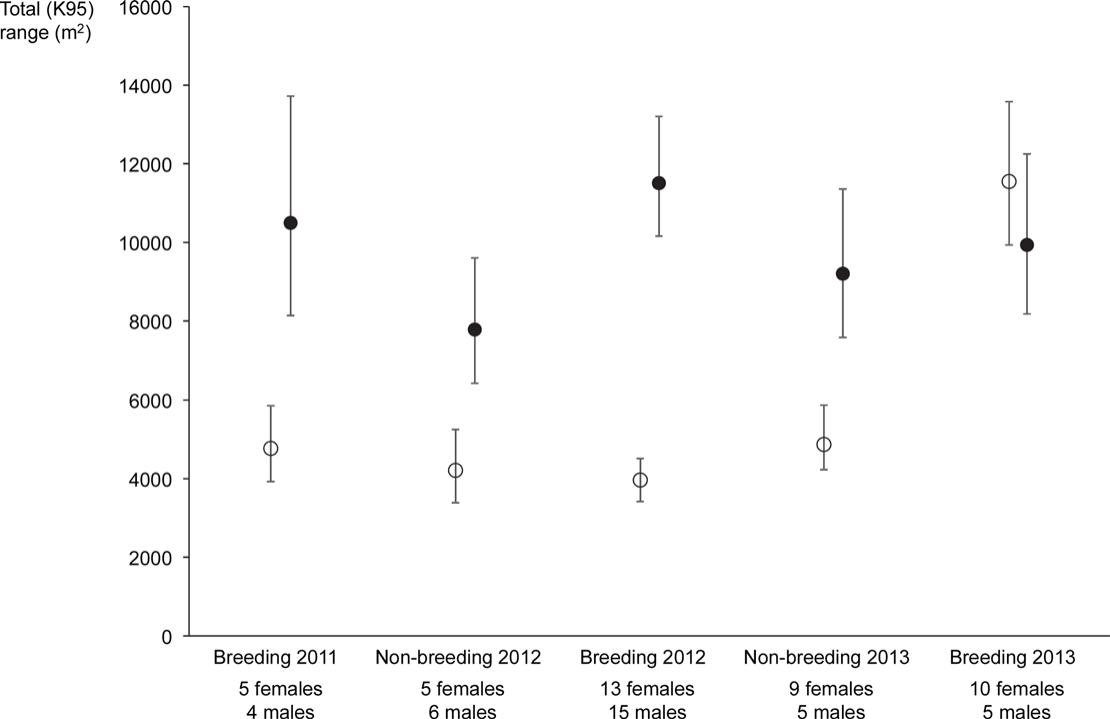
Mean predicted total home range (95% isopleth) for male (solid circle) and female (hollow circle) koalas sampled in each breeding and non-breeding season between September 2011 and November 2013. Error bars are 95% confidence intervals of the mean.

Total range size was influenced by the interaction of gender and season (S1 Table). Model predictions indicate that female total range size was relatively consistent (mean predicted range 3,840 to 4,900 m^2^) between breeding and non-breeding seasons from September 2011 –August 2013, and then increased to 10,300 m^2^ in the breeding season of 2013 (Fig 2). Mean predicted ranges of males were higher than those of females and tended to be higher in breeding than in non-breeding periods (8,750 to 11,260 m^2^ from September 2011 to August 2013). However, in contrast to the female data, mean predicted range for the 2013 breeding season was similar to that of preceding breeding seasons (8,960 m^2^; Fig 2).

**Fig 3:**
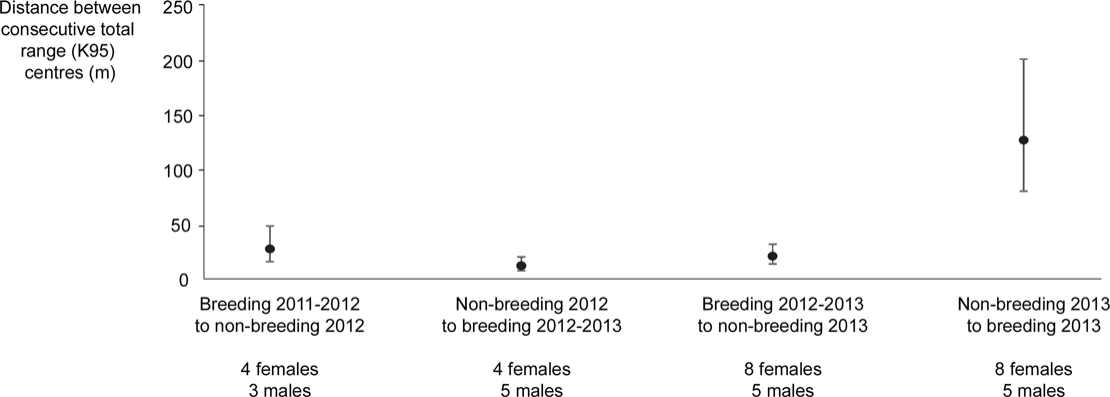
Mean predicted distance between total range centres of individuals (both sexes) in consecutive breeding and nonbreeding seasons from September 2011 to August 2013. The values in parentheses below the axis are mean UDOI ° *SE* and the number of individuals in the sample.

There was high inter-season overlap of total ranges prior to the breeding season of 2013 (mean UDOI = 0.87 ° 0.13, N = 29). For these seasons, the predicted mean distance between consecutive total range centres was only 10 to 26 m (Fig 3). There was less overlap of total ranges from the non-breeding to breeding season of 2013 (mean UDOI = 0.05 ° 0.04, N = 13) with overlap for only 23% (3/13) of individuals that were sampled in both seasons. The predicted mean distance between total range centres of these seasons increased to 144 m (Fig 3).

### Survival

Prior to the 2013 breeding season, six (22.2%; 6/27) individuals for which fate was known died. Causes were fight wounds (three males), an ecological burn in part of the site (one male, one female), and undetermined (one male). In the 2013 breeding season, 71% (15/21) individuals died or were euthanased due to poor condition. This included 75% (9/12) of females and 67% (6/9) of males across all tooth wear classes (i.e. 3.5 to 14 years of age). Condition scores were high (>7) for all but one individual at the beginning of this sampling period (September 2013), and 75% (9/12) of females had dependent young (Mean weight of young = 1.19 ° 0.21 kg). Condition scores for individuals that were euthanased (4) or retrieved within one day of death (2) were low (≤5). Eight females abandoned their young during the sampling period. Mortality of adults and abandonment of young were attributed to starvation. The six surviving koalas (3 males, 3 females) moved up to 500 m to small, isolated manna gum woodland patches where trees were not completely defoliated. Only three of these individuals showed weight loss (3.5 to 10.1% of body mass) although all showed some decline in condition score (1 to 3 point decline) between September and November 2013.

## Discussion

Our study documented the final two years of growth and subsequent catastrophic collapse of a koala population in manna gum woodland in Victoria. The browsing pressure of the high density koala population resulted in a decline in food resources over a two-year period with almost complete defoliation of manna gum across several hundred hectares of woodland by September 2013. Most radio collared koalas showed strong fidelity to small ranges prior to September 2013 and moved only short distances from their former ranges when food resources were mostly depleted. Female koalas abandoned dependent young (89%) and high mortality was observed (71.4% of radio collared koalas). The widespread nature of the problem and the large number of koalas affected prompted intervention by state government officials to euthanase starving koalas [32].

Irruptions of koala populations leading to widespread tree defoliation and mass starvation of koalas are not new in southern Australia. Such events have been observed since 1908 [33], are relatively localised and associated with the presence of preferred food trees such as manna gum and swamp gum (*E*. *ovata*). Irruptions have been recorded for naturally occurring as well as introduced or reintroduced koala populations, and across landscapes varying in terms of habitat fragmentation. Several irruptions in the early 1900s (e.g. Wilson’s Promontory [33]) occurred in patches of preferred food trees surrounded by lesser preferred eucalypt forest. More frequently, such events have occurred on islands or in mainland areas where koalas have been introduced (see [15] for review).

Our study population at Cape Otway is an introduced population founded by approximately 75 koalas that were translocated from French Island during the 1980s [32]. These koalas were released into approximately 450 hectares of manna gum dominated woodland. When we commenced our study in 2011, koala population densities were already high with an average density of 10.1 koalas per hectare across our study sites. The population then almost doubled in the next two years as a result of high fecundity and a female-biased sex ratio, low mortality, and low dispersal rates. Although fecundity estimates from surveys are only 54%, these estimates are likely to be conservative due to difficulties in detecting small joeys as well as an inability to detect pouch young. In our telemetry study, fecundity was high with ≥ 76% of females producing young in each of the 2011 and 2012 breeding seasons. Although sample size is small, mortality also appeared to be relatively low prior to September 2013 with most deaths recorded for males and as a result of fatal fight wounds.

Total home ranges documented in this study were extremely small relative to most other estimates for koalas. In the five sampling periods between September 2011 and August 2013, predicted mean total ranges were only 0.5 hectares for females and varied between 0.8 and 1.4 hectares for males. These are similar to ranges reported for other high density populations in areas of high availability of preferred food resources [34], but much lower than those in other parts of Australia (ranges of up to 300 hectares [35–37]. The small ranges we observed may reflect the high quality and relatively contiguous availability of food resources. Manna gum is known to be a preferred food tree of koalas and often supports high density koala populations [15].

It is not surprising that canopy condition declined under the browsing pressure of such a high density population. In September 2013, 71% of trees across our study sites were highly defoliated and anecdotal observations suggest that defoliation continued in subsequent months. What is surprising is that the significant depletion of food resources in 2013 failed to elicit a more effective survival response from individuals. This cannot be attributed to lack of dispersal opportunity although dispersal may have been hindered by high densities of koalas already occupying neighbouring habitats. There is relatively high connectivity of manna gum woodlands in the area with less than 200 m of open pasture between neighbouring woodlands. Furthermore, our study site was less than three kilometres from contiguous forest containing other *Eucalyptus* species eaten by koalas. Indeed, prior to September 2013, three female koalas demonstrated an ability to move large distances through this landscape. Similarly, an inability to disperse more than short distances due to poor health following the decline in resources is not supported by our study. In early September 2013, only one of 21 radio collared koalas was in poor condition which was most likely due to its old age.

We therefore suggest that failure of koalas to respond to habitat change was due to their strong fidelity to small ranges previously associated with high reproductive success and survival. Timing of food depletion to a period when females were carrying large young (Mean joey weight = 1.19 kg), and an increase in breeding behaviour in males may have contributed further to this response. The energetic cost of dispersal may be too high for females carrying large young and if mates are the resource priority for males [38] those individuals may have decided to remain close to known mates rather than to seek better food resources. Indeed, radio-collared individuals remained in close proximity to each other during the 2013 breeding season. Most females abandoned their young but this response contributed to the survival for only two of eight females.

High site fidelity extending over a number of years has been reported for koalas [34,39], even under some level of habitat disturbance [5]. Because the koala is an energy-limited species with distinct preferences for particular trees, strong site fidelity may allow individuals to optimise foraging and mating opportunities. However, like other species, site fidelity may lead to a time lag in the ability of populations to track habitat changes [40], with catastrophic consequences for individuals. This therefore raises concern for the survival of koalas throughout their range where landscapes are changing due to development, forestry practices and other disturbances.

The results of our study have important implications for management of koalas in southern Australian locations where population irruptions are likely. Because direct management of koala populations currently is limited to nonlethal methods [8], management must be implemented well in advance of population densities becoming unsustainable. As seen in our study, the animal welfare and environmental costs of not proactively managing koala populations may be considerable. Although a program was implemented in late 2013 to address animal welfare concerns at Cape Otway, only koalas that already were in poor condition were euthanased. Furthermore, the number euthanased was probably a small proportion of the population affected. Only 33% of animals that died in our study were euthanased. The long-term impacts of koala overbrowsing on the ecosystem are poorly understood but also likely to be significant. At Cape Otway, several hundred hectares of manna gum woodland were severely defoliated in 2013. Although some tree recovery is likely, this will be impacted by the surviving koala population. In our study, 28.6% of radio collared koalas survived and remained within manna gum woodland. It therefore is inevitable that without management, the koala population at Cape Otway will grow with high potential for more starvation events and eventual loss of manna gum woodland in the region.

## Supporting Information

S1 Data Koala population density, tree canopy condition and telemetry data from the study. (XLSX)

S1 Table Results of mixed model for home range size. (DOCX)
