# Supplementary material for: Failure to Respond to Food Resource Decline Has Catastrophic Consequences for Koalas in a High-Density Population in Southern Australia
Source: PLoS One. 2016 Jan 6;11(1):e0144348. doi: 10.1371/journal.pone.0144348 (PMC4703219; doi:10.1371/journal.pone.0144348)
Supplement: S1 Table — (DOCX) [file pone.0144348.s002.docx]

| **Explanatory variable** | **Estimate** | **SE** | **t-value** |
| --- | --- | --- | --- |
| **Intercept** | 8.4755 | 0.2003 | 42.32 |
| **Male^ad^** | 0.7900 | 0.3291 | 2.40 |
| **Non-breeding 2012^b^** | -0.1283 | 0.2497 | -0.51 |
| **Breeding 2012 ^b^** | -0.1993 | 0.2121 | -0.94 |
| **Non-breeding 2013 ^b^** | 0.03822 | 0.2198 | 0.17 |
| **Breeding 2013^bd^** | 0.8846 | 0.2233 | 3.96 |
| **Male; Non-breeding 2012^c^** | -0.1681 | 0.3862 | -0.44 |
| **Male; Breeding 2012^c^** | 0.2908 | 0.3452 | 0.84 |
| **Male; Non-breeding 2013^c^** | -0.1681 | 0.3885 | -0.43 |
| **Male; Breeding 2013^cd^** | -0.9389 | 0.3905 | -2.40 |

^a^ The reference category was Female

^b^ The reference category was Breeding 2011-2012

^c^ The reference category was Female and Breeding 2011-2012

^d^ Influential variables
